# Germline pathogenic variation impacts somatic alterations and patient outcomes in pediatric CNS tumors

**DOI:** 10.1101/2025.02.04.25321499

**Published:** 2025-02-06

**Authors:** Ryan J. Corbett, Rebecca S. Kaufman, Shelly W. McQuaid, Zalman Vaksman, Saksham Phul, Miguel A. Brown, Jennifer L. Mason, Sebastian M. Waszak, Bo Zhang, Chuwei Zhong, Heena Desai, Ryan Hausler, Ammar S. Naqvi, Antonia Chroni, Zhuangzhuang Geng, Elizabeth M. Gonzalez, Yuankun Zhu, Allison P. Heath, Marilyn Li, Penn Medicine BioBank, Regeneron Genetics Center, Phillip B. Storm, Adam C. Resnick, Kara N. Maxwell, Kristina A. Cole, Angela J. Waanders, Miriam Bornhorst, Suzanne P. MacFarland, Jo Lynne Rokita, Sharon J. Diskin

**Affiliations:** 1 Center for Data-Driven Discovery in Biomedicine, Children’s Hospital of Philadelphia, Philadelphia, PA, USA; 2 Division of Neurosurgery, Children’s Hospital of Philadelphia, Philadelphia, PA, USA; 3 Center for Childhood Cancer Research, Children’s Hospital of Philadelphia, Philadelphia, PA, USA; 4 Department of Biomedical and Health Informatics, Children’s Hospital of Philadelphia, Philadelphia, PA, USA; 5 Ann & Robert H. Lurie Children’s Hospital of Chicago, Chicago, IL, USA; 6 Division of Hematology, Oncology, Neuro-Oncology, and Stem Cell Transplantation, Department of Pediatrics, Northwestern University Feinberg School of Medicine, Chicago, Illinois, USA; 7 Laboratory of Computational Neuro-Oncology, Swiss Institute for Experimental Cancer Research, School of Life Sciences, École Polytechnique Fédérale de Lausanne, Lausanne, Switzerland; 8 Division of Hematology/Oncology, Department of Medicine, Perelman School of Medicine, University of Pennsylvania, Philadelphia, PA, USA; 9 Abramson Family Cancer Research Institute, Perelman School of Medicine, University of Pennsylvania, Philadelphia, PA, USA; 10 Department of Pathology and Laboratory Medicine, Children’s Hospital of Philadelphia, Philadelphia, PA, USA; 11 Penn Medicine BioBank, Department of Medicine, Perelman School of Medicine, University of Pennsylvania, Philadelphia, PA, USA; 12 Regeneron Genetics Center, Regeneron Pharmaceuticals Inc., Tarrytown, NY, USA; 13 Division of Oncology, Department of Pediatrics, Perelman School of Medicine, University of Pennsylvania, Philadelphia, PA, USA; 14 Center for Cancer and Immunology Research, Children’s National Hospital, Washington, DC, USA; 15 Department of Pediatrics, The George Washington University School of Medicine and Health Sciences, Washington, DC, USA

**Keywords:** Pediatric brain tumors, pathogenic germline variants, loss of heterozygosity, aberrant splicing

## Abstract

The contribution of rare pathogenic/likely pathogenic (P/LP) germline variants to pediatric central nervous system (CNS) tumor development remains understudied. Here, we characterized the prevalence and clinical significance of germline P/LP variants in cancer predisposition genes across 830 CNS tumor patients from the Pediatric Brain Tumor Atlas (PBTA). We identified germline P/LP variants in 24.2% (201/830) of patients and the majority (154/201) lacked clinical reporting of genetic tumor syndromes. Among P/LP carriers, 30.7% had putative somatic second hits or loss of function tumor alterations. Finally, we linked pathogenic germline variation with novel somatic events and patient survival to highlight the impact of germline variation on tumorigenesis and patient outcomes.

## Introduction

Central nervous system (CNS) tumors are the leading cause of childhood cancer death in the United States, with over 47,000 children and young adults diagnosed annually worldwide^1,2^. The overall prevalence of children with cancer with reported rare pathogenic or likely pathogenic (P/LP) germline variants in cancer predisposition genes (CPGs) ranges from 8–21%^3–10^. There are over 20 characterized genetic predisposition syndromes observed in patients with CNS tumors (Table S1) and targeted germline sequencing is being increasingly implemented in clinical settings to inform clinical practice and treatment strategies^11,12^. However, the full spectrum and prevalence of pathogenic germline variants in CPGs in patients with CNS tumors remains unknown, limiting the clinical utility of targeted testing.

The integration of germline and somatic multi-omic modalities with clinical data in pediatric cancer patients has the potential to uncover novel mechanisms underlying tumor biology and reveal clinical outcomes driven by genetic predisposition. For example, rare pathogenic germline variants in CPGs in patients with medulloblastoma have been linked to molecular subgroups, specific evolutionary trajectories, and/or biological pathway deficiencies, including chromothripsis (associated with *TP53* variants), homology-directed repair deficiency (*PALB2* and *BRCA2*), and proteome instability (*ELP1*)^13,14^.

In this study, we investigate rare pathogenic germline variants in CPGs across 830 pediatric CNS tumor patients in the Pediatric Brain Tumor Atlas (PBTA), consisting of individuals enrolled on either the Children’s Brain Tumor Network (CBTN) or a Pediatric Neuro-Oncology Consortium (PNOC) protocol^15^. We integrate multiple data modalities from matched tumors^16,17^, including DNA and RNA sequencing, proteogenomics, DNA methylation, and clinical data. We observe higher prevalence of CPG germline P/LP variants than previously reported in PBTA patients, with enrichment in PBTA relative to two cancer-free control cohorts. Importantly, we identify incidental pathogenic germline variants in pediatric CNS tumor patients, underscoring the need for implementing germline testing at diagnosis. Finally, we report new associations between rare germline pathogenic CPG variants and somatic alterations in patients with high-grade glioma, *BRAF* fusion-positive low-grade gliomas, and medulloblastoma.

## Results

### Study workflow and integrated germline-somatic analysis of pediatric CNS patients and tumors

**Figure 1.**
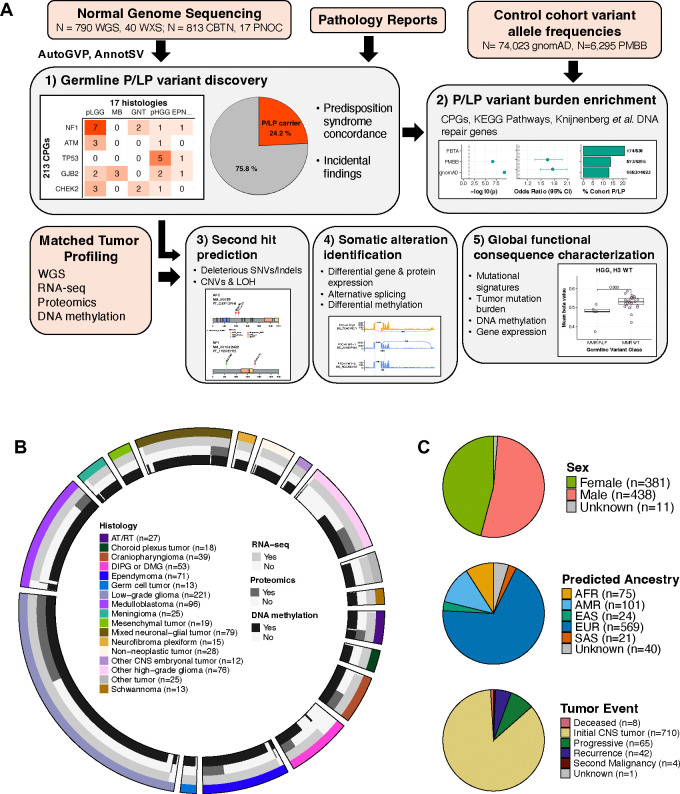
Pediatric CNS tumor cohort germline analysis summary. **A.** Overview of germline and integrative somatic analyses. **B.** Circos plot summarizing distribution of tumor histologies and availability of matched tumor RNA-seq, proteomics, and DNA methylation data modalities in 830 pediatric CNS tumor patients. Atypical Teratoid Rhabdoid Tumors (AT/RT, N=27); choroid plexus tumors (CPT, N=18); craniopharyngiomas (N=39); diffuse intrinsic pontine glioma or diffuse midline glioma (DIPG or DMG, N=53); ependymoma (EPN, N=71); germ cell tumors (GCT, N=13); low-grade gliomas (LGG, N=221); medulloblastomas (MB, N=96); meningiomas (MNG, N=25); mesenchymal tumors (N=19); mixed glioneuronal and neuronal tumors (GNT, N=79); neurofibroma plexiforms (NFP, N=15); other non-AT/RT, non-MB CNS embryonal tumors (N=12); other high-grade gliomas (HGG, N=76); and schwannomas (SWN, N=13). All other benign tumors and non-cancerous lesions were assigned to a “non-neoplastic tumor” category (N=28), and other rare CNS tumors of low sample size (N<10) were grouped into an “Other tumor” category (N=25). **C.** Sex, predicted genetic ancestry superpopulation, and matched tumor event distributions among cohort. AFR=sub-Saharan African, AMR=admixed American, EAS=East Asian, EUR=European, SAS=South Asian.

Figure 1A depicts the overall workflow of the study. Briefly, we performed germline pathogenicity assessment of rare variants (allele frequency <0.1% in all non-bottleneck populations in gnomAD non-cancer population database^18^) in 830 (N=790 WGS, N=40 WXS) pediatric CNS tumor patients with matched tumor sequencing using AutoGVP (SNV and InDels), AnnotSV, and ClassifyCNV (SVs)^19–21^, with specific focus on variants in 213 CPGs defined by an expert panel of physicians and scientists (Tables S2–S3). Germline P/LP variants were integrated with matched tumor RNA-Seq, proteogenomics, and DNA methylation array data for 770 (92.8%), 192 (23.1%), and 730 (88.0%) patients, respectively. Using 2021 World Health Organization CNS tumor classifications^22^, we categorized tumors into 15 broad histology groups (Figure 1B) and utilized molecular subtypes from the Open Pediatric Cancer Project^17^. Notably, we distinguish diffuse intrinsic pontine gliomas or diffuse midline gliomas (DIPG or DMG) from other high-grade gliomas (HGG) due to differences in anatomical location, molecular signatures, and clinical outcomes. Germline sex and genetic ancestry estimates and tumor events are shown in Figures 1C and S1. Sixty-seven patients (8.1%) had documented cancer predisposition syndromes (CPS), with the most prevalent being neurofibromatosis type 1 (NF-1) (N=25), followed by tuberous sclerosis (N=10), neurofibromatosis type 2 (NF-2) (N=7), and Li-Fraumeni syndrome (N=7) (Table S3).

### Rare P/LP germline variants in CPGs show high prevalence in pediatric CNS tumor patients

**Figure 2.**
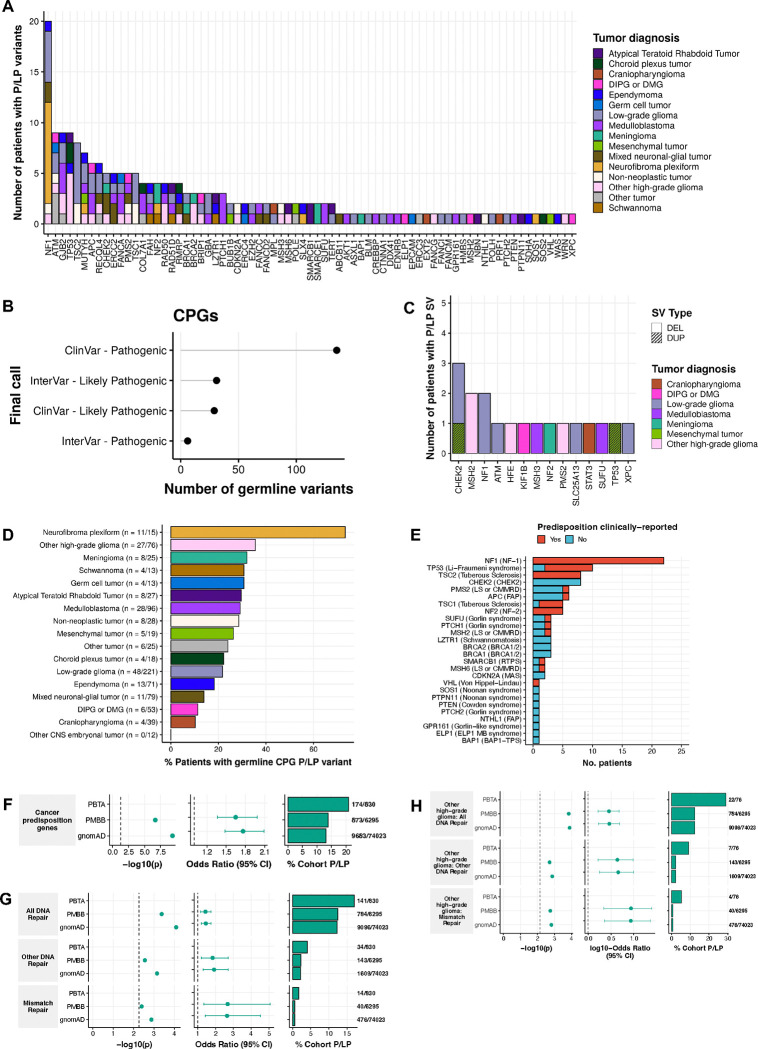
Prevalence of cancer predisposition gene germline P/LP variants in pediatric CNS tumor patients. **A.** Number of patients with identified germline P/LP variants by cancer predisposition gene (CPG) and tumor histology. **B.** Number of CPG germline P/LP SNVs and InDels identified by call and call source. **C.** Number of patients with identified P/LP structural variants by gene, tumor histology, and type. DEL=deletion, DUP=duplication. **D.** Percent of patients harboring a CPG P/LP variant by tumor histology. **E.** Number of patients harboring P/LP variants in syndrome-associated genes, and clinical definition. RTPS=Rhabdoid Tumor Predisposition Syndrome, FAP=Familial Adenomatous Polyposis, MAS=melanoma astrocytoma syndrome, BAP1 TPS=BAP1 Tumor Predisposition Syndrome. **F.** Odds ratios and associated p-values of CPG P/LP variant burden among pediatric CNS tumor cohort relative to PMBB and gnomAD cancer-free control cohorts. **G-H.** Odds ratios and associated p-values of P/LP variant burden in DNA repair genes from Knijnenburg *et al.* 2018 among **G)** entire cohort and **H)** pediatric high-grade glioma cohort (excluding DIPG and DMG) relative to control cohorts. Dashed lines in p-value plots indicate Bonferroni-adjusted p<0.05.

We identified 207 P/LP rare germline SNVs/InDels in 73 CPGs using AutoGVP^19^, among 2,815 P/LP variants across all genes (Figure 2A, Table S4A). While overall more P/LP calls were made using InterVar versus ClinVar evidence (InterVar N=1,463, ClinVar N=1,352), the majority of CPG P/LP calls had ClinVar evidence (N=169/207, 81.6%; Figure 2B, Figure S2B). As expected, we observed frameshift, missense, and nonsense variants, which are most likely to affect protein function, as the most prevalent in CPGs (30.0%, 27.1%, and 20.3%, respectively; Figure S2A). We further identified 206 germline structural variants (SVs) annotated as P/LP by AnnotSV^21^ or ClassifyCNV^20^, including 16 deletions and 2 duplications in CPGs (Figure 2C, Table S4B). There was no evidence for homozygous P/LP variants (Figure S2C); however, we identified three *MSH6* P/LP variants in a patient with HGG and confirmed constitutional mismatch repair deficiency syndrome (CMMRD; PT_3CHB9PK5), suggesting biallelic *MSH6* inactivation (Figure S2D). Another patient with medulloblastoma (MB) and no clinically-reported CPS (PT_2FK75B27) had two adjacent *PMS2* P/LP InDels that were confirmed to be on the same allele (Figure S2E).

We next sought to characterize P/LP variants in genes underlying CPSs of the CNS (Table S1). We identified 103 variants in 99 patients, of which 47 (5.7% of patients) may represent incidental findings in patients without a clinically-reported CPS (Figure 2E, Table S5). Among clinically-reported cases, we identified a corresponding germline P/LP variant in 49/57 patients (86.0%; Figure S3D). For the remaining eight cases, we expanded our variant search to those with lower variant allele frequency (0.20≥VAF≥0.08) and variants of uncertain significance (VUS), and further identified potentially deleterious variants in five cases (Table S6). These included four VUS: one *TP53* missense variant in an LFS patient (PT_PFP1ZVHD) and one *NF1* and two *NF2* splice donor variants in an NF-1 patient (PT_0FKQ3XGV) and two NF-2 patients (PT_EG7PH32E, PT_DTP4MMRA), respectively. Additionally, we identified an *NF2* pathogenic variant in an NF-2 patient with a VAF below our initial threshold (0.175). Recovering this *NF2* variant prompted us to query for other low VAF variants, and we identified nine additional CPG P/LP variants (Table S7).

Overall, CPG P/LP variants were identified in 24.2% (201/830) of patients, with 30 patients harboring two P/LP variants and two patients harboring three variants: the aforementioned CMMRD patient and a patient with a subependymal giant cell astrocytoma (SEGA, PT_JW6FBEFK) that harbored *TSC2*, *ASXL1*, and *APC* P/LP variants (Table S4A). P/LP carriers were non-randomly distributed across tumor histologies (p=5.0e-4, Table 1), and were enriched in patients with neurofibroma plexiform (N=11/15, OR=9.4, 95% CI=2.7–41.0, p=4.6e-06) and HGG (N=27/76, OR=1.9, 95% CI=1.1–3.2, p=0.02; Figure 2D, Figure S3A). We also observed P/LP carrier enrichment in patients with tumor molecular subtypes that have been previously linked to genetic predispositions (Figure S3B, Table S8). We identified CPG P/LP variants in all SEGA patients (N=10), and all but one were *TSC1* (n=3) or *TSC2* (n=6) P/LP carriers. The remaining patient carried a P/LP variant in *MUTYH*, which has not been linked to SEGA. Patients with SHH-activated MB (SHH-MB) were significantly enriched among P/LP carriers (N=12/19, OR=6.4, 95% CI=2.3–19.7, p=1.1e-04), and seven harbored P/LP variants in known SHH-MB associated genes (*PTCH1* N=3, *SUFU* N=3, *GPR161* N=1). Patients with HGG (histone H3 wildtype) were also enriched among P/LP carriers (N=16/25, OR=6.9, 95% CI=2.8–18.0, p=5.3e-06), and the majority harbored variants in mismatch repair (MMR) genes (N=6) or *TP53* (n=4). Among rare tumor types, pineoblastoma (PB) patients were significantly enriched for P/LP carriers (N=4/5, OR=13.3, 95% CI=1.3–658.4, p=0.01; Figure S3C). Interestingly, none of these patients harbored *DICER1* variants known to be associated with PB^23^, but two patients harbored *ATM* P/LP variants. Cohort-wide, we did not find any demographic features associated with P/LP carrier status (Table 1). However, among patients with mixed glioneuronal and neuronal tumors (GNTs), male P/LP carriers were overrepresented (N=10 male vs. N=1 female, OR=9.8, 95% CI=1.3–445.5, p=0.02; Table S9C).

### Germline pathogenic variation in CPGs and cancer pathways is enriched in pediatric CNS tumor patients

We assessed variant pathogenicity in non-tumor subjects from the Penn Medicine BioBank (PMBB, n=6,295) and gnomAD non-cancer samples (n=74,023) (see **Methods**). CPG P/LP variants were significantly enriched in PBTA relative to PMBB (OR=1.7, 95% CI=1.4–2.0, p=2.1e-07) and gnomAD (OR=1.8, 95% CI=1.5–2.1, p=4.4e-10) (Figure 2F, Table S10). Histology-specific analyses revealed significant CPG P/LP variant burden in patients with NF, HGG, and MB relative to controls (Bonferroni-adjusted p<0.05, OR lower 95% CI>1.0; Figure S4), *NF1*, *TSC2*, *TSC1*, *TP53*, and *NF2* had significant P/LP burden in the PBTA (Bonferroni-adjusted p<0.05, OR lower 95% CI>1.0; Figure S5A), though this was driven by the high number of patients in the cohort with LGGs, NF, and HGG. Additionally, we recapitulate previously reported histology-specific germline P/LPs in *PTCH1* for MB, *SMARCB1* for ATRT, and *SMARCE1* for meningioma (Figure S5B). Novelly, we identified significant excess *ATM* P/LP variant burden in patients with PB, although we recognize the small sample size here (N=2/5 patients).

Using rare P/LP variants in all genes, we found 13 KEGG pathways enriched in the PBTA, including five signaling pathways mediated by CPGs we previously identified as enriched (*TP53*, *TSC1*/*2*, and *NF1*). Additional significant pathways included G/S1 cell cycle regulation and cancer signaling pathways (e.g., *MTOR signaling*) (Figure S6, Table S10C). We further assessed DNA repair pathways^24^ and observed significant enrichment of P/LP variants across 1) all DNA repair genes , 2) MMR genes and 3) other DNA repair genes in PBTA (OR=1.4–2.7, 95% CI=1.2–5.0, p<1.3e-03; Figure 2G, Table S10D). This is likely driven by patients with HGG, in which we observed significant P/LP burden in DNA repair and MMR genes (OR=2.9–8.7, 95% CI=1.7–25.0, p<2.0e-03; Figure 2H) and is consistent with previous reports^16,25^.

### Germline CPG P/LP carriers exhibit loss of function (LOF) somatic events

**Figure 3.**
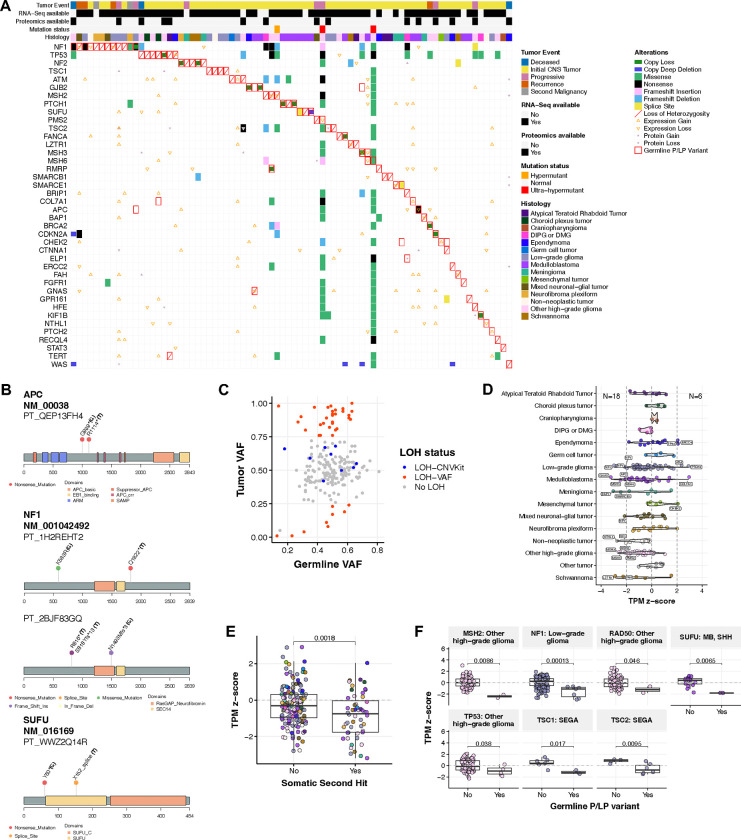
Identification of somatic second hit events in matched tumors. **A.** Oncoprint of somatic alterations (SNVs/InDels, copy number variation, loss of heterozygosity, differential gene and protein expression) in matched tumors of patients harboring CPG P/LP variants. **B.** Lollipop plots displaying germline P/LP variants (“G”) and oncogenic SNVs/InDels (“T”) in matched tumors of *APC*, *NF1*, and *SUFU* P/LP carriers. **C.** Tumor versus germline variant allele frequency (VAF) of all P/LP variants detected in matched tumors. **D.** Violin plot of cancer predisposition gene (CPG) transcripts per million (TPM) z-scores in matched tumors of CPG P/LP carriers by histology. Vertical dashed lines indicate z-score thresholds for significantly increased and decreased gene expression. **E.** TPM z-scores of CPGs in P/LP carriers with identified somatic second hits versus those with no second hits. P-value is derived from a Wilcoxon rank sum test. **F.** TPM z-scores by histology and CPG in P/LP carriers versus non-carriers, with Wilcoxon rank sum test p-values.

We assessed all matched tumors for genomic alterations indicative of LOF, including SNVs/InDels, copy number (CN) alterations, and loss of heterozygosity (LOH; Figure 3A, Table S11). We detected 204/217 (94.0%) CPG P/LP SNVs/InDels and 17/18 (94.4%) P/LP SVs in matched tumors. The 13 P/LP SNVs/InDels not found in tumors exhibited significantly lower VAF relative to those in tumors (median VAF 0.20 vs. 0.50, p=7.2e-07), suggesting that some of these may represent genetic mosaicism. Among germline P/LP variants in tumors, 69 in 56 patients harbored a putative second hit alteration.

Eight patients harbored somatic SNVs/InDels annotated as oncogenic or likely oncogenic (O/LO) by OncoKB^26^ or otherwise predicted as deleterious (see **Methods**): one *NF1* mutation in each of four unique P/LP carriers, an *APC* nonsense mutation and an *SUFU* splice site mutation in two distinct P/LP carriers with MB (PT_QEP13FH4 and PT_WWZ2QQ14R, respectively), a *MSH6* missense mutation in the P/LP carrier with CMMRD and HGG (PT_3CHB9PK5), and a *SMARCE1* splice site mutation in a P/LP carrier with meningioma (PT_NSXP8AWV; Figure 3B, Figure S7). Next, we identified 58 LOH events associated with germine P/LP variants in tumors (22.2% of all variants), of which 49 (84.5%) were associated with loss of the wildtype allele (Figure 3C). Seventeen LOH events coincided with CN loss, while the remaining 41 events occurred in CN-neutral tumors (Figure S8A). Recurrent LOH was observed for seven CPGs in five tumor histologies for which LOH has previously been reported as a mechanism for gene inactivation: *NF1* (LGG, NFP), *NF2* (MNG, SWN), *PTCH1* and *SUFU* (MB), *MSH2* (HGG), *TP53* (CPT, HGG), and *TSC1* (LGG)^13,27–29^.

To assess the potential impact of germline P/LP variants on gene expression, we surveyed transcriptional and proteomic profiles in matched tumors. We observed 18 cases of gene expression loss in matched tumors of P/LP carriers, and six cases of gene expression gain (Figure 3D). Cases with a putative somatic second hit (i.e, LOF SNV/InDel or wildtype LOH) exhibited significantly lower corresponding gene transcript abundance relative to P/LP carriers without a second hit (p=1.8e-03; Figure 3E). Recurrent transcript loss in P/LP carriers versus non-carriers was observed for seven genes across 4 cancer groups: *MSH2*, *RAD50*, and *TP53* (HGG), *NF1* (LGG), *SUFU* (SHH-MB), and *TSC1* and *TSC2* (SEGA) (Wilcoxon p<0.05, Figure 3F). Proteomics analysis of matched tumors identified three cases of protein loss in P/LP carriers: *TSC2* (PT_66XN3MT1, SEGA), *SLC25A13* (PT_MPRBGGEJ, LGG), and *ELP1* (PT_2JDDX6TJ, H3K27M DMG) (Table S11). Only the *TSC2* case showed significant transcript loss (z-score=−2.43), while the others harbored no transcript-level or somatic changes. Notably, while *ELP1* has recently been reported as a predisposition gene for SHH-MB^14^, it has not been reported as a genetic predisposition for H3K27M DMG. In summary, we identified somatic genomic or expression LOF events associated with germline P/LP variation in 66/221 (30.7%) cases.

We analyzed tumor DNA methylation data to investigate potential epigenetic changes driven by germline P/LP variation. We identified 106 hypermethylated and 337 hypomethylated probes annotated to the same gene in matched tumors of P/LP carriers relative to non-carriers (Figure S8B, Table S12). P/LP-associated hyper- and hypo-methylation were enriched in gene promoters (OR=1.9, 95% CI=1.2–2.9, p=3.2e-03) and intronic regions (OR=1.6, 95% CI=1.3–2.0, p=7.1e-05), respectively. P/LP carriers displaying promoter hypermethylation exhibited gene expression loss relative to other P/LP carriers (Figure S8C), suggesting that presence of a P/LP germline variant influences methylation patterns and subsequent gene expression in tumors. Interestingly, we observed significant *RECQL4* promoter hypermethylation at probe cg20260034 in two P/LP carriers harboring distinct variants and diagnosed with different cancers (LGG and GNT) (Figure S8D–E). No other somatic alterations were identified in these tumors, although both exhibited marginal gene expression loss (z-scores=−0.89, −1.18).

### P/LP variation is significantly associated with differential somatic alternative splicing and impacts gene expression and protein function

**Figure 4.**
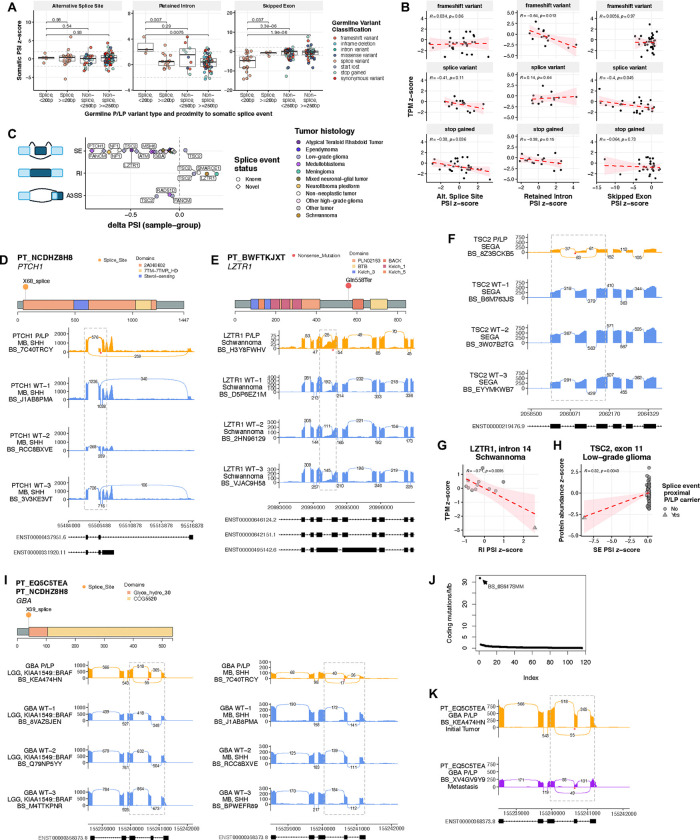
Landscape of germline P/LP variant-associated alternative splicing events in matched tumors. **A.** Germline P/LP variant-proximal splice event percent splice index (PSI) z-scores in matched tumors, grouped by proximity to germline variant and class of variant (splice vs. non-splice). P-values are derived from Wilcoxon rank-sum tests **B.** Transcripts per million (TPM) z-scores against P/LP-variant-proximal splice event PSI z-scores among frameshift variant, splice variant, and stop gained variant P/LP carriers. R values represent Pearson correlation coefficients. **C.** Significant P/LP variant-proximal alternative splicing events in matched tumors of P/LP carriers by splice event type and histology, plotted by percent spliced in (PSI) difference relative to other tumors of same histology. SE=single exon, RI=retained intron, A3SS=alternative 3’ splice site. **D.** A germline *PTCH1* splice acceptor P/LP carrier with SHH-activated medulloblastoma (SHH-MB) exhibits increased exon 2 skipping relative in matched tumor relative to other SHH-MB tumors. **E.** A *LZTR1* stop gained P/LP carrier with schwannoma exhibits increased upstream intron 14 retention in matched tumor relative to other schwannomas. **F.** A *TSC2* splice polypyrimidine tract P/LP variant carrier with subependymal giant cell astrocytoma (SEGA) exhibits increased exon 11 skipping in matched tumor relative to other SEGAs. **G.** *LZTR1* TPM z-scores against intron 14 PSI z-scores in schwannoma cases. **H.** *TSC2* TPM z-scores against exon 11 PSI z-scores in low-grade gliomas. **I.** Two *GBA* splice donor P/LP carriers with *KIAA1549::BRAF* fusion positive LGG and SHH-MB exhibit increased exon 2 skipping in matched tumors relative to other tumors of the same histology. **J.** Indexed tumor mutation burdens in *KIAA1549::BRAF* fusions-positive LGG with *GBA* P/LP carrier PT_EQ5C5TEA highlighted. **K.** *GBA* sashimi plot highlighting exon 2 skipping in initial and metastatic second malignancy of PT_EQ5C5TEA.

Due to the large number of germline splice variants in the PBTA cohort (N=37/217) and their documented impact on pediatric CNS tumor development^13,14,30^, we investigated their influence on somatic alternative splicing (AS) and subsequent functional consequences. We queried splice events (see **Methods**) in all matched tumors and observed that germline P/LP splice region variation was associated with significantly increased proximal intron retention or exon skipping relative to non-splice or distal splice P/LP variants (Figure 4A, Table S13). Strikingly, splice region variants were associated with reduced transcript and protein abundance at levels similar to those observed in frameshift and stop gained P/LP carriers (Figure S9A–B), indicating that they may elicit similar functional consequences in tumors. Frameshift and stop gained P/LP variants were associated with proximal intron retention and alternative splice site usage, respectively, each significantly negatively correlated with transcript abundance (Pearson’s R=−0.64, p=0.013 and R=−0.38, p=0.036), suggesting that such variants may impact gene expression in part through aberrant splicing events (Figure 4B).

We identified 31 significant P/LP variant-proximal AS events in matched tumors (|percent spliced in [PSI] z-score| ≥ 2) including skipped exon (SE; N=24), retained intron (RI; N=4), and alternative 3’ splice site usage (A3SS; N=3) events (Figure 4C). Intriguingly, 19 (79%) of the 24 SE splice events are not associated with splice junctions in known transcripts and are therefore considered novel. Further, we identified several tumors with gene expression loss or putative disruption of functional protein domains as defined by Pfam^31,32^ (Figure 4D–I, Figure S9C). A germline *PTCH1* splice acceptor P/LP carrier with SHH-MB (PT_NCDHZ8H8) exhibited novel somatic exon 2 skipping, and this exon encodes a portion of a conserved sterol transporter family protein domain (*2A060602*) (Figure 4D). A germline *LZTR1* stop gained P/LP carrier with schwannoma (PT_BWFTKJXT) exhibited significantly increased intron 14 retention directly upstream of this variant. This retention event was significantly negatively correlated with *LZTR1* transcript abundance in schwannoma (Pearson’s R=−0.71, p=9.5e-3; Figure 4E&G), suggesting it may be a mechanism of *LZTR1* tumor suppressor inactivation. Furthermore, a germline *TSC2* polypyrimidine tract P/LP carrier with SEGA exhibited increased exon 11 skipping that results in a novel transcript isoform (Figure 4F). This SE event was associated with significant TSC2 protein loss compared to other LGG tumors (Figure 4H). Lastly, a patient with AT/RT and a germline *LZTR1* synonymous variant exhibited increased somatic exon 4 skipping that is expected to disrupt the Kelch substrate recognition domain (Figure S9D). This variant has been shown to drive exon 4 skipping through the putative disruption of an exonic splice enhancer^33^. We observe significant LOH in this patient’s tumor, uncovering a role for *LZTR1* splice variants in AT/RT tumorigenesis that has not been previously described.

We identified two recurrent P/LP splice variants associated with proximal SE events in matched tumors: 1) P/LP *FAH* splice donor variants associated with modest but significant exon 12 skipping in LGG and schwannoma (PT_NA8NZ0BN and PT_EYWDFKA7, respectively) and 2) P/LP *GBA* splice donor variants associated with increased exon 2 skipping in SHH-MB and *KIAA1549::BRAF* fusion-positive LGG (PT_NCDHZ8H8 and PT_EQ5C5TEA, respectively) which is predicted to disrupt the glycosyl hydrolase domain (Figure 4I). Loss of GBA protein has previously been linked to increased rates of metastasis in other cancers^34,35^, and we observed that not only was PT_EQ5C5TEA’s initial tumor the most highly mutated among all *KIAA1549::BRAF* fusion-positive LGGs (31.8 coding mutations/Mb), but it was also one of only six LGGs in this cohort to metastasize (Figure 4J). This second malignancy lacked the *BRAF* fusion found in the initial tumor, but exhibited *GBA* exon 2 skipping at a higher rate, which may be due to differences in tumor purity (1.0 in second malignancy versus 0.24 in initial tumor; Figure 4K).

### Mismatch repair gene pathogenic variation drives distinct genetic and molecular signatures in pediatric high-grade gliomas

**Figure 5.**
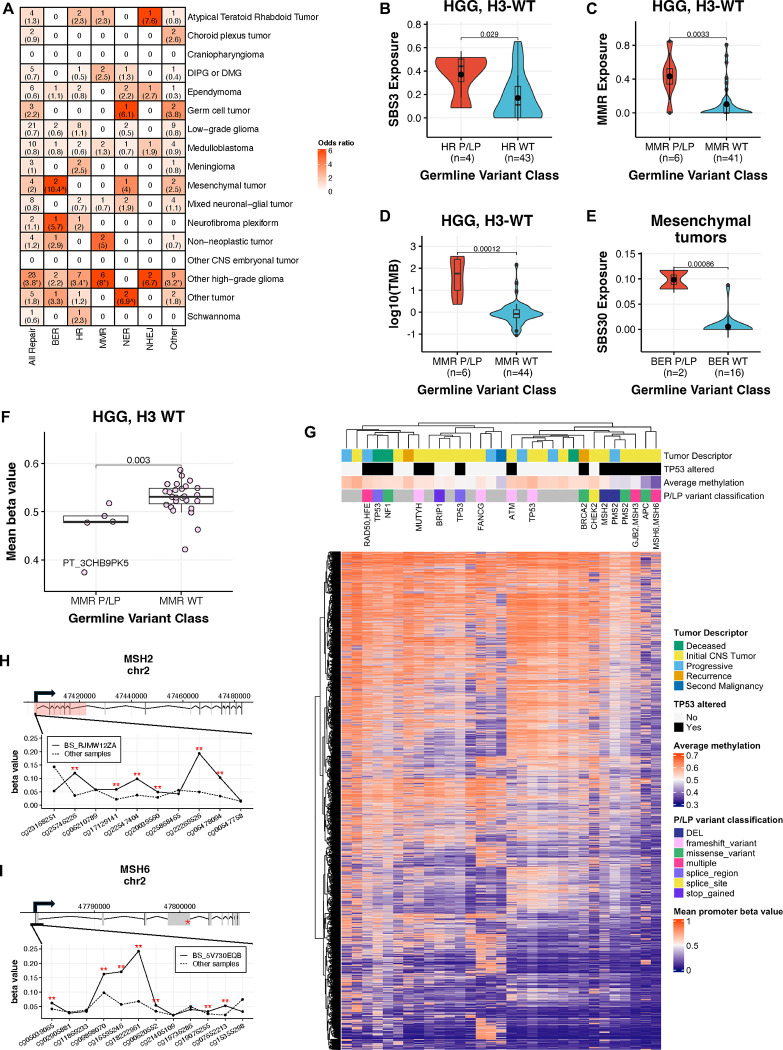
Germline P/LP variants in DNA repair genes are associated with distinct molecular profiles in high-grade glioma (HGG). **A.** Count of DNA repair variant P/LP carriers within CNS tumor histology cohorts by DNA repair pathway, with Fisher’s exact test-derived odds ratios in parentheses. Cells are colored by odds ratio weight. *=FDR<0.05, ^=p<0.05. BER=base excision repair, HR=homologous recombination, MMR=mismatch repair, NER=nucleotide excision repair, NHEJ=non-homologous end joining. “Other” indicates genes in the DNA repair list that are not in any of the five pathways. **B.** SBS3 mutational signature exposure weights in HR gene P/LP carriers versus non-carriers with H3-wildtype HGG. **C-D.** MMR deficiency mutational signatures exposure weights **(C)** and tumor mutation burden **(D)** in MMR gene P/LP carriers versus non-carriers with H3-wildtype HGG. **E.** SBS30 exposure weights in BER gene P/LP carriers versus non-carriers with mesenchymal tumors. **F.** Mean beta value from all probes on Infinium methylationEPIC array in MMR gene P/LP carriers versus non-carriers with H3-wildtype HGG, with constitutional mismatch repair deficiency (CMMRD) syndrome patient indicated by label. P-values are derived from Wilcoxon rank sum tests. **G.** Clustering of H3 wildtype HGG samples by 10k most variable promoter-annotated probes on the Infinium methylationEPIC array. Gene symbols above heatmap indicate annotation of germline P/LP variants. **H-I.** A germline P/LP deletion in *MSH2* **(H)** and P/LP SNP in *MSH6* **(I)** are associated with significant hypermethylation of respective promoter probes in H3-wildtype HGG cases. The red box and asterisk in gene models indicate positions of germline P/LP deletion and SNP, respectively. **=|beta value z-score| ≥ 2.

Given the significant P/LP variant burden in DNA repair pathways, we further explored their functional impact in tumors. We identified 97 P/LP variants across 32 DNA repair pathway CPGs in 91 (10.9%) patients (Figure S10A). HGG tumors were enriched in patients with P/LP variants in homologous recombination (HR), MMR, all repair, and other repair pathways (FDR<0.05 and OR lower CI>1 for all comparisons), while mesenchymal and other tumors were nominally enriched among base and nucleotide excision repair (BER and NER) P/LP carriers, respectively (p<0.05 and OR lower CI>1 for all comparisons; Figure 5A). We assessed COSMIC mutational signature data from these tumors and confirmed that H3-wildtype HGGs from HR and MMR gene P/LP carriers exhibited significantly higher SBS3 and MMR-deficiency signature exposures, respectively, relative to other HGGs (p=0.029 and 0.0033, Figure 5B–C, Figure S10B). This increased MMR exposure corresponded to significantly higher tumor mutation burden (TMB) compared to non-carriers (p=1.2e-04), and, consistent with previous findings in MMR-deficient HGG^36^, 4/6 tumors were considered hypermutant (>10 mutations/Mb; Figure 5D, Figure S10B, Table S14). Mesenchymal tumors with BER P/LP variants exhibited significantly increased SBS30 exposure weights relative to non-carriers (p=8.6e-4, Figure 5E). Interestingly, we observed significantly lower genome-wide mean methylation beta values in H3-wildtype HGGs from MMR P/LP carriers compared to non-carriers (p=0.003, Figure 5F). Notably, the CMMRD patient harboring three *MSH6* germline P/LP variants exhibited the lowest global CpG methylation rate. Since the tumors of MMR P/LP carriers were hypomethylated and hypermutated, we asked whether TMB and global methylation were broadly correlated in HGGs. Indeed, global methylation was significantly negatively correlated with TMB exclusively in H3 wildtype HGG tumors (Pearson’s R=−0.72, p=7.6e-06), but not in other histologies or other HGG subtypes (Figure S10C–D, Table S15). Furthermore, clustering of HGG tumors by beta values at the 20,000 most variable promoter CpGs revealed a cluster of samples distinct from heterozygous MMR gene P/LP carriers, while the CMMRD patient sample clustered separately (Figure 5G). Finally, two MMR gene P/LP carriers exhibited somatic promoter hypermethylation of the same gene, suggestive of epigenetic silencing associated with P/LP variation despite observed global hypomethylation (Figure 5H–I).

### CPG P/LP carriers exhibit distinct survival outcomes

**Figure 6.**
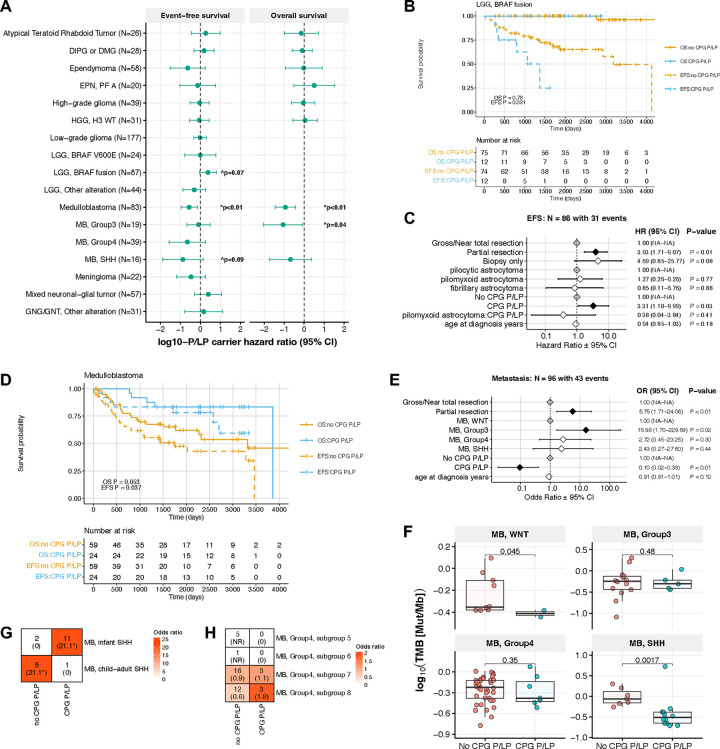
P/LP carrier status-associated survival differences in pediatric CNS tumor patients. **A.** Hazard ratios of event-free and overall survival (EFS and OS, respectively) in germline CPG P/LP carriers versus non-carriers by tumor histology or molecular subtype. Statistics were derived from cox proportional hazards models that included age at diagnosis covariate for all tumor histologies, and molecular subtype and extent of tumor resection covariates for relevant tumor histologies. **B.** Kaplan-Meier survival curves for EFS and OS in patients with *KIAA1549::BRAF* fusion-positive LGG, stratified by CPG P/LP carrier status. **C.** Cox proportional hazards model forest plot of EFS in patients with *KIAA1549::BRAF* fusion positive LGG, including covariates for extent of surgical resection, age at diagnosis, and interaction between pathology diagnosis and CPG P/LP carrier status. **D.** Kaplan-Meier survival curves for EFS and OS in patients with medulloblastoma, stratified by CPG P/LP carrier status. **E.** Logistic regression model forest plot of metastasis in patients with medulloblastoma. **F.** Tumor mutation burden in patients with medulloblastoma by CPG P/LP carrier status and molecular subtype. P-values are derived from Wilcoxon rank sum tests. **G-H.** Heatmap of patient distribution among **H)** SHH-MB subgroups and **I)** Group 4 MB methylation subgroups by CPG P/LP carrier status, with Fisher’s exact test-derived odds ratios in parentheses. *=FDR<0.05. NR=not reportable.

CPG P/LP variants have previously been associated with worse overall survival in pediatric cancers including neuroblastoma and leukemia^13,37–39^; therefore, we assessed whether P/LP carriers in the PBTA exhibited distinct survival outcomes for tumor histologies and molecular subtypes (Figure 6A, Table S16). We found that among all *KIAA1549*::*BRAF* fusion-positive LGGs, P/LP carriers exhibited significantly worse EFS versus non-carriers in a univariate model (p=0.031, Figure 6B). When accounting for the extent of tumor resection and pathology diagnosis, this trend remained significant in patients with pilocytic astrocytomas (PAs; HR=3.31, 95% CI=1.10–9.99, p=0.03; Figure 6C). The majority of P/LP carriers with PA (N=9/13) harbored variants in DNA repair genes, and, interestingly, patients with *KIAA1549::BRAF* fusion-positive LGGs exhibited the second greatest excess P/LP variant burden relative to cancer-free controls, although this was not significant after multiple test correction (Figure S11A).

Among patients with MB, CPG P/LP carriers exhibited significantly better EFS and OS relative to non-carriers (p<0.01 for both EFS and OS; Figure 6D, Figure S11B–C). To identify factors that may confer better survival among P/LP carriers with MB, we first assessed additional molecular and clinical data. We observed that MB P/LP carriers exhibited significantly lower metastasis rates (OR=0.10, 95% CI=0.02–0.4, p=2.7e-03; Figure 6E), and these were associated with worse OS (HR=10.0, 95% CI=2.2–45.3, p=2.9e-03; Figure S11D). Interestingly, we identified significantly lower TMB in WNT- and SHH-MB tumors of P/LP carriers compared to non-carriers, which has not been previously reported. In the case of SHH-MB tumors, this may be explained in part by earlier ages at diagnosis observed in P/LP carriers (Figure 6F, Figure S11E). We further delineated SHH-MB tumors and observed that infant SHH (β and γ) tumors were enriched in P/LP carriers (N=6, OR=1.7, 95% CI=1.4–1395.7, p=0.01; Figure 6G), while child-adult SHH (α and δ) tumors were enriched in non-carriers and exhibited worse OS and EFS (Figure S11F). Similarly, we only identified P/LP carriers with subgroup 7 (N=3/19) and 8 (N=3/15) Group 4 MBs and these patients had significantly better EFS and OS than non-carriers with subgroup 5 tumors, though we acknowledge the low numbers in these groups. (Figure 6H, Figure S11G). In summary, P/LP carriers with MB Group 4 subgroups 7 or 8, infant-type SHH MB, and/or MB with decreased metastasis confer better overall survival.

## Discussion

This study represents the largest survey of germline pathogenic variation in a pediatric pan-CNS tumor data set to our knowledge. We identified germline CPG P/LP variants in 201/830 (24.2%) patients, a higher frequency than has been reported in previous germline studies (8–21%) which may be due, in part, to the larger CPG list utilized here^3–5,7,8,13^. While the majority of CPG P/LP SNVs/InDels called by AutoGVP utilized ClinVar evidence, we identified 37 additional P/LP variants through AutoGVP’s modified InterVar classification, 18 P/LP SVs and one deep intronic *NF1* variant previously classified as P/LP^40^, highlighting the importance of leveraging multiple approaches to characterize the full spectrum of disease-causing germline variation in pediatric cancers. We observed high concordance between clinically-reported CPSs and prevalence of associated CPG P/LP variants, and identified several VUS in patients lacking P/LP variants that warrant further study into their pathogenicity. The majority of identified germline P/LP variants were absent from clinical records, including a high proportion of variants in genes linked to CPSs. Several factors may contribute to this, such as the extended timeframe of sample collection (spanning over 13 years), the non-routine nature of germline testing, and the evolving understanding of CPSs. These findings underscore the importance of increasing routine germline testing in pediatric CNS tumor patients to better guide monitoring and treatment strategies even in the absence of family history. For example, we identified several patients with germline P/LP *TP53* or MMR gene variants with no clinically reported CPS; the former would benefit from lifetime monitoring for early detection of secondary cancers as is routine for patients with LFS, and the latter could receive immunotherapies given the prevalence of chemoresistant tumors^41^. While the majority of histology-specific P/LP CPGs have been previously described, we observed that 2 of 5 patients with pineoblastoma harbored an *ATM* germline P/LP variant. To our knowledge, *ATM* has not been implicated in development of pineoblastoma, although future studies are needed to assess their prevalence in a larger cohort.

We detected a putative somatic second hit (LOF SNV/InDel, LOH) in tumors of 28.6% (N=56/196) of P/LP carriers, suggesting that observed pathogenic germline variation in these patients is indeed contributing to tumorigenesis. While representing roughly one-third of cases, and within the range of second hit frequencies reported in previous pediatric cancer studies (10–55%)^3–5,8,37^, there may be additional somatic inactivating mutations in functionally-related genes and/or pathways, and/or haploinsufficiency alone may initiate tumorigenesis^42^ in some cases. Somatic SNVs/InDels in the same P/LP gene were rare, while CN-neutral LOH resulting in loss of wildtype allele was pervasive, particularly in *NF1*, *TP53*, *PTCH1*, *MSH2*, and *TSC2*.

We report transcriptomic, regulatory, and proteomic somatic alterations indicative of gene inactivation, many in cases in which a tumor DNA second hit was not observed. The majority of somatic differential gene expression in CPG P/LP carriers indicated loss of transcript in the same gene, though there were rare cases of gene expression gain. We further identified splice region P/LP variants driving somatic aberrant splicing and disruption of protein domains with subsequent gene expression and protein loss. Beyond splice region variants, we report cases of frameshift or stop gained variant-associated AS, indicating that splicing may contribute to LOF driven by these variant classifications as has been reported previously^43^. Intriguingly, we identify a recurrent *GBA* splice donor P/LP variant associated with increased exon 2 skipping in a *BRAF*-fusion positive LGG with high TMB and a metastatic event. While *GBA* variants are a risk factor for Gaucher Disease, and altered *GBA* expression has been linked to increased tumor malignancy^34,35^, the relationship between *GBA* germline variation and LGG tumorigenesis and/or malignancy has not been studied. Our work emphasizes the need for further investigation into the contributions of AS to pediatric CNS tumor development and progression.

Lastly, we observed global functional consequences of germline MMR gene P/LP variation in patients with histone H3-wildtype HGG, including increased MMR-deficiency mutational signatures, TMB, and global hypomethylation, the latter of which has not been reported previously. Strikingly, we found increased TMB to be associated with global hypomethylation exclusively in H3-wildtype HGG, but not in other HGG subtypes nor in other tumor histologies.

Germline CPG pathogenic variation was associated with distinct clinical outcomes in a subset of patients. CPG P/LP carriers with *BRAF* fusion-positive PAs exhibited worse EFS compared to non-carriers. Interestingly, P/LP carriers with PA diagnoses disproportionately harbored DNA repair gene variants, which were also nominally enriched among *BRAF*-fusion positive LGGs relative to control populations. Future research should seek to assess the contribution of DNA repair gene germline pathogenic variation to *KIAA1549::BRAF* LGG tumorigenesis. Conversely, CPG P/LP carriers with MB exhibited better survival relative to non-carriers, which may be explained by lower metastasis rates and TMB, earlier ages at diagnosis, and clinically favorable methylation subgroups associated with P/LP carriers in this MB cohort. While inactivation of known MB predisposition genes has previously been associated with increased metastasis risk^44,45^, this has not been explored in the context of germline variation to our knowledge. Future studies with a larger cohort are required to assess unique germline variant-associated tumor characteristics that may contribute to clinical outcomes in MB. In summary, our study has identified new functional links between germline variation, tumor molecular features, and clinical outcomes in pediatric CNS tumor patients, highlighting the importance of routine germline testing at diagnosis. Continued understanding of germline susceptibility and its influence on tumorigenesis will aid in identification of patients that may benefit from genetic counseling, surveillance, altered treatment regimens, and ultimately clinical outcomes.

## Online Methods

### Pediatric CNS tumor/normal samples and germline SNV/indel calling

The cohort used in this study is composed of patients from the Children’s Brain Tumor Network (CBTN; n=813) and the Pediatric Neuro-Oncology Consortium (PNOC; n=17). Germline variant calls from whole genome sequencing (WGS) data for paired tumor (~60X) and normal peripheral blood (~30X)^16,17^ whole exome sequencing (WXS) data for paired tumor (~470X) and normal peripheral blood (~308X) were obtained through CBTN and PNOC data access requests. Briefly, paired-end WGS reads were aligned to the version 38 patch release 12 of the Homo sapiens reference genome using BWA-MEM^46^. The Broad Institute’s Best Practices^47^ were used to process Binary Alignment/Map files (BAMs) in preparation for variant discovery. Duplicate alignments were marked using SAMBLASTER^48^, and merged and sorted BAMs using Sambamba^49^. The BaseRecalibrator submodule of the Broad’s Genome Analysis Tool Kit (GATK)^50^ the GATKHaplotypeCaller^51^ submodule to generate a genomic variant call format (GVCF) file. This file was used as the basis for germline calling. Germline haplotype calling was performed using the GATK Joint Genotyping Workflow on individual gVCFs from the germline sample alignment workflow. The GATK generic hard filter suggestion was applied to the VCF (SNPs only), with an additional requirement of 10 reads minimum depth per variant. This Kids First workflow can be found at https://github.com/kids-first/kf-germline-workflow.

### Germline variant annotation and assessment of variant pathogenicity

Resulting variants were annotated using the Kids First Data Resource Center (KFDRC) Germline SNV Annotation Workflow and added the following annotations: ENSEMBL 105, ClinVar (2022May07), and InterVar (https://github.com/kids-first/kf-germline-workflow/blob/master/docs/GERMLINE_SNV_ANNOT_README.md). Germline variants in 213 cancer predisposition genes were further analyzed.

Pathogenicity was assessed for filtered variants *in silico* using AutoGVP to evaluate ClinVar (2022May07) and a modified execution of InterVar^19^. We applied a pathogenicity pre-processing workflow on VEP-annotated VCFs that encompasses variant annotation with ANNOVAR (database pulled on 2024May06), InterVar v2.2.1 variant classification, and AutoPVS1 v2.0.0 variant scoring. Variants with read depth coverage ≥ 10, variant allele fraction ≥ 0.20, and observed in <0.1% across non-bottleneck gnomAD v3.1.1 non-cancer populations (African/African American, Admixed American, non-Finnish European, South Asian, East Asian) were retained for variant classification. Pathogenicity assessment was performed using AutoGVP as previously described^19^. Briefly, we pulled classifications for rare variants annotated in the ClinVar database with ≥2 stars or with 1 star and no conflicting submissions. All variants with conflicting classifications in ClinVar were resolved by first filtering for submissions that have been associated with MedGen disease concept IDs, and taking the single submission or consensus submission if available. Remaining variants classifications were resolved by taking the classification at the last date evaluated. For remaining variants without ClinVar annotation, we adjusted PP5 based on this modified InterVar assessment and corrected PVS1 using AutoPVS1. Variants were assigned as pathogenic (P), likely pathogenic (LP), benign (B), likely benign (LB), or unknown significance (VUS) by first considering ClinVar results and then the modified InterVar output. This approach was applied to pediatric CNS cases and non-cancer control samples (PMBB and gnomAD 3.1.1). For variants with conflicting submissions in ClinVar that were resolved as non-P/LP, we reviewed cases for which there was a P or LP submission that provided functional evidence, and upgraded the classification accordingly when such evidence was sufficient.

All cases of proximal adjacent InDels in a single patient and InDels ≥ 20 bp were manually reviewed in Integrated Genomics Viewer (IGV) and removed if variants were not present. We also queried samples for intronic NF1 variants identified in Koczkowska et al. 2023^40^ that were shown to associate with alternative splicing events and classified these as LP.

### Germline structural variant calling and pathogenicity assessment

We applied the KFDRC Germline Structural Variant Caller Workflow to generate SV pathogenicity calls from normal sequencing data (https://github.com/kids-first/kf-germline-workflow/blob/master/docs/GERMLINE_SV_README.md). Briefly, Manta v1.6.0^52^ was run on all aligned paired-end sequencing reads to call SVs, and AnnotSV v3.1.1^21^ was run on Manta output to annotate SV pathogenicity. We filtered out any P/LP SVs present in >1% of the PBTA cohort, and any that overlapped with SV or CNV annotated in gnomAD at an allele frequency > 0.01% in a non-bottleneck population.

### Cohort demographics and clinical characteristics

Genetic ancestry prediction was performed on all patients with normal WGS data as previously described^53^. Differences in demographic and clinical characteristics between subjects with and without germline P/LP variants in CPGs were assessed across the cohort and within cancer histologies. Fisher’s exact tests were used to compare categorical data variables (sex, race, ethnicity, predicted genetic ancestry) and Mann Whitney-U tests were used to compare numerical data variables (age at diagnosis, overall and event-free survival, tumor mutation burden). A two-sided Fisher’s exact test or Mann Whitney-U test p-value <0.05 was considered significant.

### CPG P/LP Enrichment

The gnomAD v3.1.1 and PMBB cancer-free control cohorts were run through the same pathogenicity preprocessing and assessment pipelines. The PMBB cohort consisted of tumor-free patients without a family history of cancer, as defined previously^37^. Variants identified in the PMBB cohort were subjected to the same manual curation used for the PBTA cohort. Enrichment of P/LP germline variants across all CPGs and within individual CPGs in the PBTA cohort vs. control cohorts was performed using Fisher’s exact tests. The same statistical framework was applied to assess enrichment of P/LP germline variants among genes in KEGG pathways and DNA repair pathways as defined in Knijnenburg et al 2018^24^, regardless of whether the gene was also defined as a CPG. Bonferroni adjustment was performed for gene and pathway analyses to correct for multiple tests. Enrichment of CPGs and pathway P/LP variants was also run within tumor histology cohorts using the same methodology.

### Somatic second hit analyses

Somatic SNVs, CNVs, and SVs were obtained from the Open Pediatric Cancer (OpenPedCan) Project release v15^16,17^. Somatic mutations in CPGs were annotated with oncoKB to identify those classified as oncogenic or likely oncogenic (O/LO)^26^. We defined somatic SNV/indel second hits as those defined as those annotated as O/LO or otherwise classified as “probably_damaging” or “possibly_damaging” by PolyPhen or “deleterious” by SIFT. Copy number variants (CNVs) were identified in tumor samples as previously described^16^ based on consensus among Control-FREEC^54^, CNVkit^55^, and GATK^50^. We assessed LOH in matched tumors using two methods: 1) comparison of VAFs in tumor vs. germline, and 2) estimating tumor allele copy number using CNVkit^55^, the latter of which accounts for low tumor purity which limited detection of LOH by VAF in some cases. We created the publicly-available tool AlleleCouNT (https://github.com/d3b-center/AlleleCouNT)^56^ to obtain germline and tumor allele counts of each CPG P/LP variant in P/LP carriers, and calculated odds ratios of variant frequencies using Fisher’s exact tests. Loss of heterozygosity (LOH) affecting the wildtype allele was defined by one of the following criteria: 1) tumor VAF > 0.75, odds ratio > 1 and Fisher’s exact p < 0.05, or 2) CNVkit copy number of wild type allele=0. LOH affecting the variant allele was defined by the following criteria: 1) tumor VAF < 0.25, odds ratio < 1 and Fisher’s exact p < 0.05, or 2) CNVkit copy number of variant allele=0. We calculated gene-level LOH score using the following equation across all rare variant showing evidence of heterozygosity in the germline:

gene_LOH_score=∑|variant_AF_tumor-0.5|

Genes were defined as exhibiting significant LOH if gene_LOH_score≥0.25.

### Gene Expression

RNA-seq data were obtained from the OpenPedCan release v15^17,57^. Z-scores were calculated using the formula z=(x-μ)=σ where x is the sample TPM for a given gene, μ is the mean gene TPM across samples, and σ is the TPM standard deviation. We considered a gene as differentially expressed relative to other samples of the same tumor histology when |TPM z-score| ≥ 2.

### Alternative Splicing

We queried replicate Multivariate Analysis of Transcript splicing (rMATs) files from OpenPedCan release v15^17,57^ for the most proximal splice event of each case type (skipped exon [SE], retained intron [RI], and alternative 3’ and 5’ splice site [A3SS and A5SS, respectively] to each CPG P/LP variant, and calculated percent spliced in (PSI) value z-scores for these events in each P/LP carrier using the same method we applied to TPM values. Since rMATs only reports splice events in a sample that are either supported by 1) the provided gene annotation file or 2) sample RNA-seq reads, we classified each splice event as known (i.e., annotated in a transcript) or novel (i.e., not found in any annotated transcript). In the case of novel SE and RI events that were not reported in a given sample, we set PSI equal to 1 for SE events (indicating no evidence for exon skipping) and 0 for RI events (indicating no evidence for intron retention) to obtain the most accurate assessment of differential alternative splicing in P/LP carriers versus non-carriers. In cases where multiple exon skipping events were reported for the same exon but with different flanking exons, we retained the event with the greatest PSI difference in the P/LP carrier versus non-carriers. Significant germline P/LP variant-associated differential splicing events were defined as events with |PSI z-score| ≥ 2 and |PSI sample - mean PSI| > 0.05, where the P/LP variant was <250bp from the splice junction.

### DNA Methylation

DNA methylation array beta values were obtained from OpenPedCan release v15^17^. To determine if P/LP carriers exhibited somatic differential methylation of affected CPG, we calculated beta value z-scores of each probe associated with that CPG in the same manner as described for gene expression. Significant differential methylation was defined as |sample beta value z-score| ≥ 2 and |sample beta value - mean beta value| > 0.05. We also calculated mean beta values across all measured probes for each patient as an estimate of relative global methylation across individuals.

### Proteomics

Proteomics data from the Clinical Proteomic Tumor Analysis Consortium (CPTAC PBTA, pediatric) and Project HOPE (adolescent and young adult HGG) were obtained from OpenPedCan release v15^17,58^. Sample-level protein abundance z-scores in P/LP carriers were calculated relative to other samples of the same histology were calculated as previously described. Significant differential protein abundance was defined as |sample protein abundance z-score| >= 2.

### Tumor mutational signatures analysis

Mutational signatures analysis was performed using the deconstructSigs^59^ R package function <monospace>whichSignatures(),</monospace> input consensus SNV file, and COSMIC v3.3 mutational signatures database as described in the Open Pediatric Cancer Project^17^. We performed Mann Whitney-U tests to assess differences in mutational signature exposure weights between samples with and without germline P/LP variants in DNA repair. Exposure weight differences were plotted using ggplot2 <monospace>geom_bar()</monospace> function^60^. Differences in mutational signatures exposure weights in >2 groups were assessed using one-way analysis of variance (ANOVA) and post-hoc pairwise comparisons, and were plotted using ggplot2 <monospace>geom_violin()</monospace> function^60^.

### Survival analyses

We performed Kaplan-Meier analyses of overall and event-free survival (OS and EFS, respectively) to compare outcomes of patients with and without germline P/LP variants in CPGs. Patient events that were included in EFS calculations were as follows: tumor progression, recurrence, and second malignancy, and death due to disease. We generated Cox proportional-hazards regression models to identify variables that were predictive of outcome. Variables considered included presence of germline P/LP variant in a CPG, molecular subtype, and extent of tumor resection (low-grade glioma only). The cohort analyzed included all those for whom data were available for all variables in the model (n=654 for OS, n=652 for EFS). Survival analyses were run using the survival R package^61,62^.

## Supplementary Material

Supplement 1

Supplement 2

Supplement 3

Supplement 4

Supplement 5

Supplement 6

Supplement 7

Supplement 8

Supplement 9

Supplement 10

Supplement 11

Supplement 12

Supplement 13

Supplement 14

Supplement 15

Supplement 16

Supplement 17

## Figures and Tables

**Table 1.** Demographic and clinical features of PBTA cohort by CPG P/LP carrier status

	CPG P/LP	No CPG P/LP	p

**Number Cohort**	201 (24.2%)	629 (75.78%)	

**Sex**			0.35
Female	99 (49.3%)	282 (44.8%)	
Male	101 (50.2%)	337 (53.6%)	
Unknown	1 (0.5%)	10 (1.6%)	

**Race**			0.27
American Indian or Alaska Native	0 (0%)	5 (0.8%)	
Asian	2 (1%)	25 (4.0%)	
Black or African American	14 (7.0%)	53 (8.4%)	
More Than One Race	2 (1%)	9 (1.4%)	
Native Hawaiian or Other Pacific Islander	0 (0%)	1 (0.2%)	
Other/Unknown Race	37 (18.4%)	98 (15.6%)	
White	146 (72.6%)	438 (69.6%)	

**Ethnicity**			0.70
Hispanic or Latino	23 (11.4%)	61 (9.7%)	
Not Hispanic or Latino	170 (84.6%)	537 (85.4%)	
Other/Unknown Ethnicity	8 (4.1%)	31 (4.9%)	

**Genetic Ancestry Superpopulation**			0.72
AFR	18 (9.0%)	57 (9.1%)	
AMR	30 (14.9%)	71 (11.3%)	
EAS	4 (2.0%)	20 (3.2%)	
EUR	139 (69.2%)	430 (68.4%)	
SAS	5 (2.5%)	16 (2.5%)	
Unknown	5 (2.5%)	35 (5.6%)	

**Tumor Histology**			***5.0E-04***
ATRT	8 (4.1%)	19 (3%)	
Choroid plexus tumor	4 (2.0%)	14 (2.2%)	
Craniopharyngioma	4 (2.0%)	35 (5.6%)	
DIPG or DMG	8 (4.0%)	45 (7.2%)	
Ependymoma	13 (6.5%)	58 (9.2%)	
Germ cell tumor	4 (2.0%)	9 (1.4%)	
Low-grade glioma	50 (24.9%)	171 (27.2%)	
Medulloblastoma	29 (14.4%)	67 (10.7%)	
Meningioma	8 (4.0%)	17 (2.7%)	
Mesenchymal tumor	5 (2.5%)	14 (2.2%)	
Mixed neuronal-glial tumor	11 (5.5%)	68 (10.8%)	
Neurofibroma plexiform	11 (5.5%)	4 (0.6%)	
Non-neoplastic tumor	8 (4.0%)	20 (3.2%)	
Other CNS embryonal tumor	0 (0%)	12 (1.9%)	
Other high-grade glioma	27 (13.4%)	49 (7.8%)	
Other tumor	6 (3.0%)	19 (3.0%)	
Schwannoma	5 (2.5%)	8 (1.3%)	

**Median age at diagnosis, years**	7.96	8.45	0.52
**Median tumor mutation burden**	0.39	0.36	0.11

## Data Availability

All pediatric brain tumor raw data are available upon request from the database of Genotypes and Phenotypes (dbGAP), accession number phs002517.v4.p2, or from the Children’s Brain Tumor Network (https://cbtn.org) and the Pediatric Neuro-Oncology Consortium (pnoc.us). All processed somatic data used in this study were derived from the OpenPedCan project^17^ v15 data release^63^ at https://github.com/d3b-center/OpenPedCan-analysis. All original code and manuscript figures and tables are available at https://github.com/diskin-lab-chop/pbta-germline-somatic.
